# Pasture enclosures increase soil carbon dioxide flux rate in Semiarid Rangeland, Kenya

**DOI:** 10.1186/s13021-018-0114-4

**Published:** 2018-12-07

**Authors:** Collins O. Oduor, Nancy Karanja, Richard Onwong’a, Stephen Mureithi, David Pelster, Gert Nyberg

**Affiliations:** 1 Department of Land Resource Management and Agricultural Technology (LARMAT), University of Nairobi, P. O. Box 29053-00625, Nairobi, Kenya; 2 Mazingira Centre, International Livestock Research Institute, P. O. Box 30709-00100, Nairobi, Kenya; 3 Agriculture and Agri-Food Canada, Science and Technology Branch, Quebec City, Canada; 4 Department of Forest Ecology and Management, Swedish University of Agricultural Sciences (SLU), 90183 Umea, Sweden

**Keywords:** Carbon dioxide, Methane, Nitrous oxide, Vegetation cover, Soil respiration, Pastoral ecosystem

## Abstract

**Background:**

Pasture enclosures play an important role in rehabilitating the degraded soils and vegetation, and may also influence the emission of key greenhouse gasses (GHGs) from the soil. However, no study in East Africa and in Kenya has conducted direct measurements of GHG fluxes following the restoration of degraded communal grazing lands through the establishment of pasture enclosures. A field experiment was conducted in northwestern Kenya to measure the emission of CO_2_, CH_4_ and N_2_O from soil under two pasture restoration systems; grazing dominated enclosure (GDE) and contractual grazing enclosure (CGE), and in the adjacent open grazing rangeland (OGR) as control. Herbaceous vegetation cover, biomass production, and surface (0–10 cm) soil organic carbon (SOC) were also assessed to determine their relationship with the GHG flux rate.

**Results:**

Vegetation cover was higher enclosure systems and ranged from 20.7% in OGR to 40.2% in GDE while aboveground biomass increased from 72.0 kg DM ha^−1^ in OGR to 483.1 and 560.4 kg DM ha^−1^ in CGE and GDE respectively. The SOC concentration in GDE and CGE increased by an average of 27% relative to OGR and ranged between 4.4 g kg^−1^ and 6.6 g kg^−1^. The mean emission rates across the grazing systems were 18.6 μg N m^−2 ^h^−1^, 50.1 μg C m^−2 ^h^−1^ and 199.7 mg C m^−2 ^h^−1^ for N_2_O, CH_4_, and CO_2_, respectively. Soil CO_2_ emission was considerably higher in GDE and CGE systems than in OGR (*P* < 0.001). However, non-significantly higher CH_4_ and N_2_O emissions were observed in GDE and CGE compared to OGR (*P* = 0.33 and 0.53 for CH_4_ and N_2_O, respectively). Soil moisture exhibited a significant positive relationship with CO_2_, CH_4_, and N_2_O, implying that it is the key factor influencing the flux rate of GHGs in the area.

**Conclusions:**

The results demonstrated that the establishment of enclosures in tropical rangelands is a valuable intervention for improving pasture production and restoration of surface soil properties. However, a long-term study is required to evaluate the patterns in annual CO_2_, N_2_O, CH_4_ fluxes from soils and determine the ecosystem carbon balance across the pastoral landscape.

## Background

The increased mean global temperatures currently experienced is associated with the increasing atmospheric concentration of greenhouse gasses (GHG) over the last century [1]. Globally, land use change and forestry, and agriculture accounts for about 10.0% and 11.2% of total anthropogenic GHG emissions, respectively [2]. Kenya’s GHG emissions in 2015 were estimated to be 30 million tons of carbon dioxide equivalent (MtCO_2_e) and is projected to rise to 39 MtCO_2_e by 2030 unless appropriate mitigation actions are taken [3]. The agriculture sector contributes approximately 41% of total anthropogenic GHG emissions [4]. Pastoralism is the dominant land use and the most important economic and livelihood activity in the 85% of Kenya’s land area classified as arid and semi-arid (ASAL) [4]. At the same time, the livestock sub-sector is reported to contribute over 50% of Kenya’s agricultural GHG emissions [5]. The vastness of ASALs coupled with poor grazing management has exacerbated the contribution of the livestock sub-sector to the national GHG inventories. Whereas open grazing management has caused soil and vegetation degradation [6], the establishment of pasture enclosures through fencing of communal grazing land is a restoration technique commonly practiced in rangelands [7–9].

Unlike exclosure management systems where livestock grazing is prohibited, livestock-based pasture enclosures were introduced in West Pokot County in Kenya, as a management tool to rehabilitate the degraded communal/open grazing lands [10]. The enclosures are private grazing areas which have been physically fenced-off to avoid interference by the rest of the community and livestock for a certain period (usually three years) to allow natural regeneration of plants [11]. According to Wairore et al. [12], grazing dominated enclosure (GDE) and contractual grazing enclosure (CGE) are the common types of enclosure management systems in Chepareria, in West Pokot County. Contractual grazing represents a grazing arrangement where a farmer owning few animals leases the enclosure to households with relatively more livestock. On the other hand, the GDE system is where the livestock utilizing the enclosure are purely owned by the farmer. The enclosures are privately owned with an average size of 5 ha and a stocking rate ranging between 1 and 42 (with a mean of 7) animals [12]. Livestock management in both CGE and GDE systems is through the free-range system of grazing. The pasture enclosures in Chepareria have been reported to enhance the soil quality in terms of particulate organic carbon and microbial biomass contents [13]. Research in northern Ethiopia suggests that vegetation properties, like species diversity and ground cover within enclosures, improve with the age of enclosures [14, 15].

Degraded soils often have low GHG emission rates [16], and restoration of these soils may increase the emission of GHGs [17]. The increased GHG emissions from restored rangelands are thought to be related to the increased vegetation cover and biomass production [7, 18], soil organic carbon (SOC) content [9], improved soil moisture content [7], and the reduced soil compaction [19]. Plant biomass contributes to soil organic matter which may increase the rate of soil respiration and organic matter mineralization, emitting CO_2_ to the atmosphere [20, 21]. Raich and Schlesinger [22] concluded that root respiration and decomposition of organic matter are the main sources of CO_2_ emission from the soil. Mineralization of soil organic matter also leads to accumulation of ammonium and nitrates thereby stimulating nitrification and denitrification processes [23], which contribute up to 70% of the global N_2_O emissions [24]. Dung (or manure) from grazing animal remains to be the major source of CH_4_ in rangelands [25, 26]. The effect of grazing on bio-chemical processes that influence GHG emissions may vary with the type of grazing management practice. For example, high concentrations of nutrients and microorganisms in vegetated sites may increase GHG emission compared to bare soil, with soil moisture strongly regulating the fluxes [27–29]. Unger et al. [30] reported that the drying and wetting cycles in soil stimulates microbial respiration rate, though respiration declined naturally by 40% within a few hours after wetting. Generally, microbial respiration is considered the largest source of atmospheric CO_2_ in the carbon cycle [31].

However, no study in Kenya and in East Africa has conducted direct measurements of GHG fluxes in the following the restoration of degraded communal grazing lands through the establishment of pasture enclosures. Furthermore, the previous study was conducted in exclosures in the temperate grasslands of central Tibetan Plateau in China [32], suggesting a distinct lack of data on the response of GHG fluxes following the establishment of pasture enclosures in West Pokot County. To address this gap in the knowledge, measurements of key GHG fluxes (CO_2_, CH_4_, and N_2_O) were carried out in the pasture enclosures and in the adjacent open rangeland as the control. The aims of the study were to investigate; (1) the effect of pasture enclosures on the emission rates CO_2_, CH_4_ and N_2_O, and (2) the seasonal variation of the key GHG fluxes and their relationships with surface soil and vegetation factors (soil organic carbon, soil moisture, vegetation cover and aboveground biomass). This study was based on the hypothesis that higher GHG flux rates were expected to occur in the pasture enclosure than in the open grazing rangeland.

## Materials and methods

### Site description

**Fig. 1:**
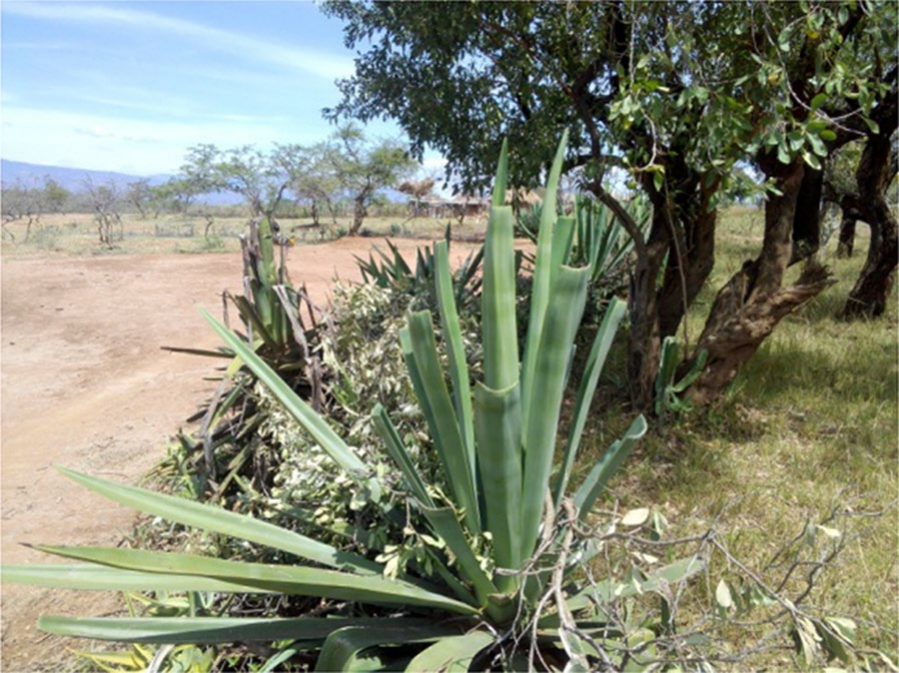
A pasture enclosure and open grazing land in the adjacent in Chepareria, Kenya

The study was conducted in Yuwalteke location in West Pokot County, in Kenya, during the dry season and long rainy season of 2017. Yuwalteke is located within Chepareria Ward on the lower slopes of Kamatira hills (between latitude 1°18′–1°19′N and longitude 35°14′–35°15′E) at an altitude of 1560 meters above mean sea level. The area is classified as semi-arid (Agroecological zone IV); receiving on average 280 mm of rainfall for the short rains which occur between mid-October and January and 570 mm for the long rains which occur between mid-March and July [33]. The maximum (30 °C) and minimum (16 °C) air temperatures occur in the months of February and July, respectively. The soils are predominantly sandy clay and are classified as Haplic Lixisols [34]. Detailed soil characteristics of the study area are described in [35]. The main land-use and source of livelihood in the area is predominantly agro-pastoralism [36]. The area had a history of severe land degradation prior to the establishment of the enclosures [11] (Fig. 1).

### Selection of enclosures and sampling strategy

In consultation with local leaders and officials from Vi-Agroforestry, 18 enclosures were selected from CGE and GDE based on three age classes; 3–10, 11–20, and > 20 years since establishment with three replications in each age class (n = 3). The adjacent open grazing rangeland (OGR) was considered as the control (n = 9), giving a total of 27 sampling plots. Within each grazing system, three 50 m long transects were laid out in a Z-shaped orientation 10 m from the edge to avoid edge effects. Along each transect, five sampling points were marked at 10 m interval where soil and vegetation samples were collected.

#### Sampling of vegetation and analysis

Sampling of vegetation was conducted once at the peak of the short rain season (November 2016) to represent the vegetation characteristics in the grazing systems and during the subsequent measurement of greenhouse gasses. Point-to-line transect method [37], was used to assess herbaceous vegetation cover and aboveground biomass. Within each grazing system, three 50-m transects were laid in a Z-shaped orientation 10 m away from the edge. Transects were assessed using the point quadrat method as described by Daget and Poissonet [38]. A long metallic wire that was sharpened on one end was descended from the transect to the ground to make the point. A total of 100 points were made per transect at 50 cm intervals. At each of the 100 points, vegetation type (i.e., grass, forb, or shrub), or ground cover (bare ground) that intersects the point was recorded as a "hit". The vegetation and bare ground covers were estimated using Eq. 1. Above-ground biomass was assessed using a 0.25 m^2^ quadrat that was laid at intervals of 10 m along the transect giving a total of five sampling points per transect. Grass and forbs within the quadrat were clipped at 2 cm above the ground level, the fresh weight determined then oven-dried in the laboratory to a constant weight at 70 °C for 72 h.

1 $$\mathrm{Vegetation cover}= (n/N)$$

where: *n* = the number of hits of all plant species or type of ground touched, *N* = the total number of hits (100 hits in this case).

#### Soil sampling and analysis in the laboratory

Soil samples were collected within the 0.25 m^2^ quadrat after clipping the grass and forb materials. Five samples were collected per transect at 10 m intervals using a hand auger at 0–10 cm. Soil samples from each transect were mixed to form three composite samples in each age-based class and open grazing system. The samples were analyzed for pH, electrical conductivity (EC), soil total porosity, total soil organic carbon (SOC), total nitrogen (TN) and soil bulk density (BD). Soil pH and EC were determined in soil–water suspension (1:2.5 weight/volume). Soil pH was measured using a glass electrode pH meter model (HI 2211, Hanna instruments), while EC was measured using a conductivity meter model (HI 9812, Hanna Instruments). Soil total porosity was calculated using an estimated particle density of 2.65 g cm^−3^. The SOC concentration was determined using the Walkley–Black wet oxidation method [39] and TN concentration was determined using the Kjeldahl method [40]. Cation exchange capacity (CEC) was determined by the ammonium acetate (NH_4_OAc) method as described by Chapman [41]. Steel cylinders of 98.2 cm^−3^ were used to obtain undisturbed soil samples for soil bulk density determination using the same sampling design [42]. The SOC, TN, and BD were used for assessing the relationship between soil parameters and GHG flux rate.

### Gas sampling and laboratory analysis

Field gas measurements were conducted between 29 January and 28 February 2017 for the dry season and between 13 April and 13 May 2017 for the wet season. At each sample location, 3 static opaque frames measuring 27 cm × 37.2 cm × 10 cm were installed at least 5 cm deep 2 months prior to the first sampling, and remained in place throughout the study period. Sampling was conducted once a week for 4 weeks during the dry season and twice a week for 2 weeks during the wet season, making a total of eight sampling dates. Sampling time was between 9.00 and 15.00 h. To cancel the effect of time, the last sampling point was the first sampling point in the subsequent sampling event, and vice versa. On each sampling date, a lid (27 × 37.2 × 12.5 cm) fitted with a reflecting tape at the top, a rubber sealing, a fan, a 50 cm non-forced vent, a thermometer (model Einstich—TFA) and a sampling port, was fitted to the frame using metal clamps for 30 min. Four gas samples were taken at 10 min intervals (0, 10, 20, and 30 min). A 20 ml sample was drawn from each of the three chambers using a 60 ml syringe at each time interval, mixed and then the pooled sample was transferred into 20 ml pre-evacuated glass vial [43]. The CO_2_, CH_4_ and N_2_O concentrations were analyzed within 24 h at the Mazingira Centre (at the International Livestock Research Institute, Nairobi, Kenya) using a gas chromatograph (8610C; SRI, Santa Monica, CA) equipped with a flame ionization detector for CH_4_ and CO_2_ (after being methanized) and a ^63^Ni electron capture detector for N_2_O. The CO_2_, CH_4_, and N_2_O concentrations in the samples were calculated based on the peak areas measured by the gas chromatograph relative to the peak areas measured from calibration gasses. The GHG flux rates were calculated using linear regression of gas concentrations versus chamber closure time and corrected for temperature and moisture, using Eq. 2 outlined in Jiang et al. [44].

2 $$F=\frac{P}{Po}*\frac{M}{Vo}*\frac{dc}{dt}*\frac{To}{T}*H$$

where *F* is the flux rate in mg C m^−2 ^h^−1^ for CO_2_, μg C m^−2 ^h^−1^ for CH_4_ and μg N m^−2 ^h^−1^ for N_2_O; *P* is the atmospheric pressure of the sampling site (Pa); *M* is the gas mass (g mol^−1^); *dc*/*dt* is the rate of concentration change; *T* is the absolute chamber temperature at sampling time (°C); *Vo*, *Po*, and *To* are the molar volume, atmospheric pressure, and absolute chamber temperature, respectively (ml, Pa, and °C), under standard conditions; and *H* is the chamber height over the soil surface (cm).

Air temperatures (TA) at 1.5 m above ground and inside the chamber (TC) were measured simultaneously in each gas sampling event using digital probe thermometer (Einstich—TFA). Soil moisture content (SM, %v/v) and soil temperature (T_S_) were measured at 5 cm depth using soil moisture and temperature sensor model 5MT, Decagon Devices Inc. Soil moisture was converted to water-filled pore space (WFPS) using the bulk density using Eq. 3 as outline in Zhang et al. [45].

3 $${WFPS}=\left( \frac{{{volumetric}\;{moisture}\;{content}\, (\%)}}{{\left(1-\left(\frac{BD}{2.65}\right)\right)}}\right)$$

where BD is soil bulk density (g cm^−3^) and 2.65 is soil particle density of quartz (g cm^−3^).

### Statistical analysis

Shapiro–Wilkes test for normality was performed on CO_2_, CH_4_ and N_2_O flux rates at *P * ≤ 0.05. The effects of the enclosure type and age on total SOC, vegetation cover, biomass production, and GHG flux rates were analyzed by two-way ANOVA using GenStat, 14th edition [46]. Means were separated using Fischer’s protected least significant difference (LSD) test, with differences considered significant at *P* ≤ 0.05. Multiple linear regression analysis was conducted using SPSS version 20.0 [47] to determine the factors which influence GHGs emission rate where SOC, total nitrogen, soil moisture, soil temperature, soil bulk density, vegetation cover, and aboveground biomass were considered the independent factors.

## Results

### Vegetation cover and biomass under the three grazing systems

Total herbaceous vegetation cover was on average 1.8 times higher in CGE and GDE than in the OGR while aboveground biomass was 6–8 times in CGE and GDE than in the OGR (Table 1). Perennial grass cover dominated in GDE whereas annual grasses and forbs cover were high in OGR and CGE respectively. Generally, perennial grass cover and total herbaceous vegetation cover increased with the age of enclosure but the differences between the age classes was not significant (Table 2). However, no interaction was observed between type of enclosure and age class for all the parameters (Table 2). However the age of enclosure did not 178 affect annual grass or forbs cover (*P* > 0.05).

**Table 1:** Vegetation cover and biomass of the three grazing systems in Chepareria, Kenya

	Grazing systems			LSD	cv%	P-value
	OGR	CGE	GDE			
Bare ground (%)	79.27 ± 2.64a	65.58 ± 5.97b	59.78 ± 5.48c	2.01	7.10	< 0.001
Perennial grasses (%)	2.89 ± 1.48c	7.84 ± 4.49b	13.44 ± 3.57a	1.37	30.8	< 0.001
Annual grasses (%)	14.44 ± 2.45a	7.71 ± 1.67c	11.91 ± 2.75b	0.98	20.7	< 0.001
Forbs (%)	3.40 ± 2.21c	18.87 ± 2.96a	14.87 ± 7.05b	1.17	22.6	< 0.001
Total plant cover (%)	20.73 ± 2.64c	34.42 ± 5.97b	40.22 ± 5.48a	2.01	15.1	< 0.001
Herbaceous aboveground biomass (kg DM ha^−1^)	72.0 ± 54.7c	483.1 ± 170.0b	560.4 ± 193.1a	61.10	39.4	< 0.001

Values are means ± standard deviation (SD) (n = 9). Different lowercase letters indicate significant differences between grazing systems (*P* < 0.05)

*OGR* open grazing rangeland, *GDE* grazing dominated enclosure, *CGE* contractual grazing enclosure

**Table 2:** Effect of enclosure age on herbaceous vegetation cover and aboveground biomass in Chepareria, Kenya

Enclosure system	Age class (years)	Bare ground (%)	Perennial grass (%)	Annual grass cover (%)	Forbs (%)	Total plant cover (%)	Aboveground biomass (kg DM ha^−1^)
GDE	3–10	59.10 ± 1.6	12.3 ± 0.9	12.0 ± 0.6	19.2 ± 0.8	40.9 ± 1.6	474.7 ± 50.1
	11–20	61.13 ± 1.5	13.7 ± 0.9	11.3 ± 0.9	18.7 ± 0.8	38.9 ± 1.5	593.3 ± 56.5
	> 20	59.07 ± 1.1	14.3 ± 0.9	12.4 ± 0.6	18.7 ± 0.8	40.9 ± 1.1	613.3 ± 36.3
CGE	3–10	65.1 ± 1.5	5.9 ± 1.1	8.0 ± 0.4	15.1 ± 0.8	34.9 ± 1.5	406.7 ± 34.6
	11–20	68.3 ± 1.6	7.7 ± 1.2	7.2 ± 0.5	15.2 ± 0.9	31.7 ± 1.6	520.0 ± 48.9
	> 20	63.3 ± 1.3	10.0 ± 1.1	7.9 ± 0.4	14.3 ± 0.7	36.7 ± 1.3	522.7 ± 42.8
LSD_0.05_		4.085	2.844	1.666	2.218	4.085	128.1
*P*-value		0.61	0.514	0.92	0.82	0.61	0.95

Values are means ± standard deviation (SD) (n = 9)

*GDE* grazing dominated enclosure, *CGE* contractual grazing enclosure

### Soil properties

Soil pH and CEC were consistent across all the grazing systems (Table 3, *P* > 0.05). Total soil organic carbon and nitrogen concentrations were significantly higher in GDE and CGE than in OGR, with the corresponding C:N ratio exhibiting a similar trend (Table 3). The OGR system had significantly higher soil bulk density and lower total porosity than in GDE and CGE (Table 3).

**Table 3:** Soil characteristics (0–10 cm) of three grazing systems in Chepareria, Kenya

Grazing system	pH	SOC (g/kg)	TN (g/kg)	C:N	CEC (cmol_(+)_/kg)	BD (g/cm^3^)	Porosity (%)
GDE	6.1 ± 0.56a	6.6 ± 0.87a	0.7 ± 0.08a	10.2 ± 1.33a	8.7 ± 1.03a	1.4 ± 0.06b	46.4 ± 1.81a
CGE	6.2 ± 0.22a	6.2 ± 0.78a	0.6 ± 0.08a	10.0 ± 1.30a	8.9 ± 0.78a	1.4 ± 0.05b	45.4 ± 2.09b
OGR	6.0 ± 0.27a	4.9 ± 0.69b	0.5 ± 0.07b	9.2 ± 1.30b	8.9 ± 0.87a	1.5 ± 0.05a	44.8 ± 1.84b
LSD_0.05_	0.215	0.441	0.434	0.724	0.478	0.017	0.640
cv%	6.2	13.7	13.2	13.5	9.9	3.2	3.8
*P*-value	0.36	< 0.001	< 0.001	0.03	0.70	< 0.001	< 0.001

Values are means ± SD (n = 9). Different lowercase letters indicate significant differences between grazing systems (*P* < 0.05)

*SOC* soil organic carbon, *TN* total nitrogen, *C:N* carbon to nitrogen ratio, *CEC* cation exchange capacity, *BD* bulk density, *OGR* open grazing rangeland, *GDE* grazing dominated enclosure, *CGE* contractual grazing enclosure

### Soil moisture, air and soil temperature, and water filled pore space

Air temperature ranged from 25.2 to 28.6 °C while soil temperature varied between 31.5 and 38.1 °C, and both exhibited significant seasonal variations (Tables 4, 5). Soil moisture (SM) ranged between 7.2 and 11.8% (v/v) during the dry season and 16.8 and 20.9% (v/v) during the wet season in all the grazing systems, and was consistently higher in GDE and CGE than in OGR (*P* < 0.001) (Tables 4, 5). The corresponding WFPS was also higher in GDE and CGE than in OGR (*P* < 0.001) and varied between 10.2–31.9 and 29.0–52.1% during the dry and wet seasons respectively (Tables 4, 5).

**Table 4:** Soil and air conditions under the three grazing management systems during the study period

	Grazing system	Season	
		Dry	Wet
Air temperature (°C)	GDE	28.55 ± 0.35	25.31 ± 0.66
	CGE	28.48 ± 0.36	25.31 ± 0.33
	OGR	27.97 ± 0.42	25.20 ± 0.77
Soil temperature (°C)	GDE	38.13 ± 0.68a	31.52 ± 0.90
	CGE	37.06 ± 0.87a	31.79 ± 0.64
	OGR	35.39 ± 0.90b	31.67 ± 1.42
Soil moisture (% v/v)	GDE	11.77 ± 1.11a	20.89 ± 0.64a
	CGE	9.78 ± 0.99ab	19.55 ± 0.56a
	OGR	7.16 ± 1.12b	16.76 ± 0.87b
Water filled pore space (%)	GDE	25.87 ± 2.45a	46.01 ± 1.43a
	CGE	21.44 ± 2.19ab	43.07 ± 1.26ab
	OGR	16.81 ± 2.73b	38.39 ± 2.00b

Values are seasonal means ± SE (n = 9). Different lowercase letters indicate significant differences among grazing systems for each parameter (*P* < 0.05)

*OGR* open grazing rangeland, *GDE* grazing dominated enclosure, *CGE* contractual grazing enclosure

**Table 5:** Two way analysis of variance tables for soil air and soil temperatures, soil moisture and water-filled pore space (WFPS)

	Air temperature	Soil temperature	Soil moisture	WFPS
Grazing system	0.773	0.376	< 0.001	< 0.001
Season	< 0.001	< 0.001	< 0.001	< 0.001
Grazing system*season	0.891	0.299	0.924	0.888

### Emission of greenhouse gasses from the soil

**Fig. 2:**
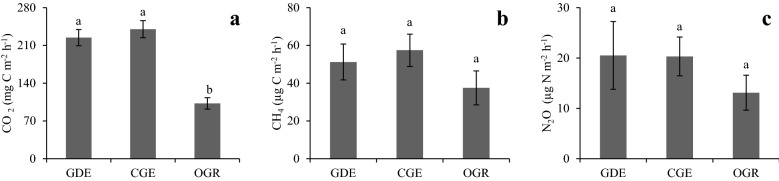
Mean emission of soil CO_2_ (**a**), CH_4_ (**b**), and N_2_O (**c**) in Chepareria, Kenya. *GDE* grazing dominated enclosure, *CGE* contractual grazing enclosure, *OGR* open grazing rangeland. Different lowercase letters denote significant differences between the grazing systems. Error bars represent standard error of the mean (SE)

**Fig. 3:**
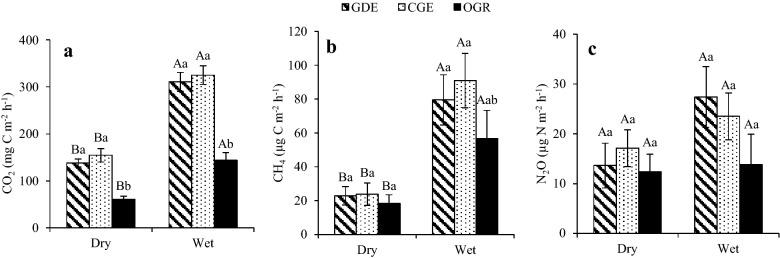
Seasonal emission of soil CO_2_ (**a**), CH_4_ (**b**), and N_2_O (**c**) in Chepareria, Kenya. *GDE* grazing dominated enclosure, *CGE* contractual grazing enclosure, *OGR* open grazing rangeland. Different uppercase and lowercase letters denote differences between seasons and the grazing systems respectively. Error bars represent standard error of the mean (SE)

The mean (± SE) soil CO_2_ flux rates in CGE (239.9 ± 15.8) and GDE (224.4 ± 15.0) were significantly (*P* < 0.001) higher compared to OGR (102.4 ± 10.6) (Fig. 2a). However, the difference in soil CO_2_ flux rate between the CGE and GDE was not significant. Significant interaction was exhibited between grazing system and season with higher CO_2_ emissions observed during the wet season in all the grazing systems (*P* = 0.02, Fig. 3a). Relative to the minimum and maximum CO_2_ emission in the OGR, the minimum and maximum CO_2_ emission in CGE and GDE were higher by 186.3 and 32.1% and 298.7 and 41.5% respectively, implying that GDE substantially increased soil CO_2_ emission. Generally, the soil CO_2_ emission rate increased with the age of enclosure and was 209.2 ± 17.5, 234.5 ± 18.8 and 252. 7 ± 19.9 mg C m^−2 ^h^−1^ in the 3–10, 11–20 and > 20 years age classes respectively, although the differences were not significant (*P* = 0.27) (Table 6).

**Table 6:** Greenhouse gas flux rates in the enclosure age classes in Chepareria, Kenya

Enclosure system	Age class (years since establishment)	CO_2_, mg C m^−2 ^h^−1^	CH_4_, μg C m^−2 ^h^−1^	N_2_O, μg N m^−2 ^h^−1^
GDE	3–10	186.0 ± 22.8	34.9 ± 8.2	32.4 ± 18.9
	11–20	226.3 ± 21.7	63.1 ± 16.3	9.5 ± 2.4
	> 20	260.9 ± 31.1	55.6 ± 17.9	18.95 ± 6.6
CGE	3–10	232.4 ± 26.2	60.8 ± 12.9	17.5 ± 5.9
	11–20	242.7 ± 31.2	53.3 ± 14.6	26.2 ± 6.9
	> 20	244.6 ± 25.6	58.0 ± 21.1	17.8 ± 7.4
LSD_0.05_		74.60	43.91	26.59
*P*-value		0.50	0.52	0.25

Values are means ± SE (*n* = 3). Different lowercase letters indicate significant differences among grazing systems (*P* < 0.05)

*GDE* grazing dominated enclosure, *CGE* contractual grazing enclosure

The CGE and GDE had higher emission rates of CH_4_ and N_2_O than OGR; but the differences between the grazing systems were not significant (*P* = 0.29 and 0.58 for CH_4_ and N_2_O respectively) (Fig. 2b, c). Higher CH_4_ and N_2_O emission rate were observed during the wet season than dry season in all the grazing systems, however this was only significant (*P* < 0.001) for CH_4_ emission (Fig. 3b, c). Similar to the CO_2_ emission rate, the age of enclosure did not influence CH_4_ and N_2_O flux rates (Table 6).

### Relationship between greenhouse gas fluxes and environmental parameters

Soil moisture exhibited significant positive correlation with GHG flux rates (*P* < 0.001); with peak emission rates were observed at soil moisture content between 15 and 25% (v/v). This relationship was stronger for CO_2_ compared to CH_4_ and N_2_O (Table 7), *R*^*2*^ = 0.10, 0.15 and 0.39 for N_2_O, CH_4,_ and CO_2_ respectively. In addition, CO_2_ emission rate showed significant positive relationship with organic carbon and above-ground biomass (Table 7).

**Table 7:** Relationship between GHG flux rates and the environmental parameters under the grazing systems (n = 216)

	CO_2_			CH_4_			N_2_O		
	Coeff.	Std. error	P-value	Coeff.	Std. error	P-value	Coeff.	Std. error	P-value
*Intercept*	− *275.8*	*235.55*	*0.01*	*14.9*	*161.58*	*0.03*	− *166.94*	*96.94*	*0.05*
Soil organic carbon	34.03	16.31	*0.04*	17.47	11.19	0.12	4.13	6.71	0.54
Total nitrogen	− 123.1	136.37	0.37	− 239.73	93.54	0.06	− 80.92	56.12	0.15
Bulk density	137.15	139.82	0.33	113.54	95.91	0.24	110.24	57.54	0.06
Soil temperature	0.39	1.43	0.78	− 1.71	0.98	0.08	0.4	0.59	0.49
Soil moisture	10.6	1.16	*< 0.001*	3.35	0.8	*< 0.001*	1.9	0.48	*< 0.001*
Total herbaceous vegetation cover	− 2.52	2.91	0.39	− 2.96	2	0.14	0.73	1.2	0.54
Above ground biomass	0.17	0.08	*0.03*	0.07	0.05	0.17	− 0.03	0.03	0.38

## Discussions

### Effect of pasture enclosures on vegetation cover and aboveground biomass

The higher herbaceous vegetation cover, perennial grass cover and above-ground biomass production in GDE and CGE demonstrated that rehabilitation of degraded grazing land occurred after enclosing the area and reducing the grazing intensity. This may be attributed to the reduced grazing pressure in the pasture enclosures relative to open grazing sites which allowed time for natural regeneration of plants. According to Mekuria and Veldkamp [48], free grazing and human interference in open grazing lands affect the regeneration and growth of herbaceous vegetation. In addition, low herbaceous plant cover and high soil compaction in OGR lead to high loss of soil water via runoff and evaporation could have reduced the availability of water to plants causing drought-induced mortality of non-woody plants [49]. Our finding corroborates with previous studies, which reported that continuous grazing in communal grazing lands reduced herbaceous cover [7, 18, 50].

The high SOC content and low bulk density in enclosed systems indicated that soil physicochemical properties were improved following the establishment of enclosures; consequently, plant growth and regeneration were enhanced. Higher perennial grasses cover than annual grasses and forbs covers in GDE suggest that lower grazing pressure supported the growth and regeneration perennial grasses. A study in China's grasslands reported that lowering grazing intensity in an overgrazed grassland allowed regeneration of desirable grass species [51]. The non-significant effect enclosure age on annual grass and forbs cover was consistent with studies conducted in southern Ethiopia and in northwestern Bolivia [52, 53]. This was because annual grasses and forbs dominated across the enclosure age classes. This explains the higher cover of perennial grass in the older (> 20 years) enclosures which also contributed to the higher biomass production in the same age class.

### Effect of pasture enclosures on surface soil properties

The improved soil properties in the enclosure compared to open grazing area indicated the potential of pasture enclosures to restore degraded soils in semi-arid rangelands. Higher SOC and TN in CGE and GDE may be due to the increased litter input in the surface 0–10 cm of soil as a result of the enhanced production of aboveground biomass. This is supported by the high C/N ratio in CGE and GDE relative to OGR which reflected a higher input of decomposable organic matter in the restored grazing areas. The results corroborated studies which attributed the increased concentration of SOC to high litter input [54, 55]. Furthermore, the higher vegetation cover in GDE and CGE relative to OGR could have reduced the loss of SOC in the topsoil via erosion. Lal [56] and Lal et al. [57] reported that wind erosion contributes to a considerable loss of SOC in the soil surface in arid and semi-arid grazing lands. Similarly, Wu et al. [58] reported that soils in degraded communal grazing land have less organic C and N compared to soils in the restored areas. Reduced trampling by livestock and higher organic carbon content in GDE and CGE contributed to the decrease in soil bulk density in the pasture enclosures relative to OGR. The non-significant difference in total organic C concentration among the enclosure age-classes and between GDE and CGE supports the studies which acknowledged that it requires several years to detect changes in total SOC [59]. As reported by Xu et al. [60] restoration of severely degraded sandy grassland is a slow process, contributing the observed similarity of soil pH and CEC in all the grazing systems in Chepareria.

### Effect of pasture enclosures on GHG emissions from soil

The mean CO_2_ flux rate in the pasture enclosures (232.2 mg C m^−2 ^h^−1^) was somehow comparable to CO_2_ flux rate recorded agricultural soils in Kenya and Tanzania (> 200 mg C m^−2 ^h^−1^) [57], but higher than those recorded in a grazed alpine steppe in China (ranged between 92.7 and 156.1 mg C m^−2 ^h^−1^) [32]. The study in China was conducted under temperate and humid conditions characterized by short summers and long cold winters, mean annual temperature ranged from − 1.5 to 2.5 °C. The relatively higher temperatures in tropical rangelands enhanced soil respiration which resulted in increased CO_2_ emission. Besides, soils in this study are well drained and may have contributed to the high diffusion rate of CO_2_ from the soil to the atmosphere. The higher emission rate of CO_2_ in GDE and CGE than in OGR was attributed to the high SOC and soil moisture content in the enclosures which increased respiration activities of soil microbes. This is supported by the positive relationship that CO_2_ exhibited with SOC and soil moisture. Also, the high above-ground biomass in the enclosure systems could mean that the below-ground root biomass was equally high [61]. Consequently, autotrophic respiration of plant roots increased the emission of CO_2_ in the enclosures than in the OGR. In contrast, previous studies in degraded rangelands either reported that restoration reduced or had no impact on soil respiration [62–65]. However, our results were consistent with studies which showed that the establishment of enclosures on previously degraded semi-arid grassland increased the emission of CO_2_ from soil [66, 67]. The high CO_2_ flux rate in the older enclosures (> 20 years), could be due to the dominance of perennial grasses which have greater root biomass than annual grasses and forbs and produce more root exudates and substrates [67], which supported microbial respiration activities in soil.

The maximum CO_2_ emission rate occurred at WFPS between 25 and 55%. Below the 25% WFPS, soil respiration was inhibited by limited soil moisture content. On the other hand, WFPS above 55% reduced soil respiration by the lowing the availability of in the soil oxygen as most of the soil pores was filled with water. Thus slowing down the decomposition of organic matter, and reduced the diffusion of CO_2_ into the atmosphere [68]. The significant positive relationship which soil CO_2_ exhibited with the SOC, soil moisture, and above ground biomass implies that availability of soil organic matter substrates and soil moisture status are the key factors influencing soil respiration in the area. The high retention of soil moisture in GDE and CGE than in OGR as instigated by the rainfall events, explains the observed seasonal variation in the emission rate of CO_2_ from the soil. These observations were consistent with previous studies which showed that soil moisture and soil organic carbon content are important factors controlling soil CO_2_ emission in grazing lands [22, 68–70]. These findings corroborate with studies which reported enhanced soil CO_2_ emission in vegetated sites compared to degraded bare soils [26, 71], and that soil respiration increased with increasing soil moisture and SOC content [72, 73].

Although CH_4_ and N_2_O uptakes (negative fluxes) were recorded in all the grazing systems, the mean flux rates were positive indicating that the grazing systems acted as net sources for atmospheric CH_4_ and N_2_O. As much as aerobic soils are widely regarded as sinks for atmospheric CH_4_ [16, 74, 75], results in this study show that mean CH_4_ flux rates in all the grazing systems were positive. This implies that soils in the grazing lands of Chepareria emit CH_4_ to the atmosphere, contrary to most agricultural soils in East Africa [76]. Since the measurements of GHGs were conducted under natural field conditions with livestock grazing activities going on, the measured CH_4_ could have been released from the traces of animal manure that were deposited within the chambers and in the surrounding. Moreover, the surface soil bulk density in this study was generally higher than that those reported in some pasture lands in Kenya and Tanzania [64]. This indicated that soils were relatively compacted and hence the availability of anaerobic microsites with low redox potential that supported the activity of methanogens, as observed by Samal et al. [25]. Despite the similarity in CH_4_ emission rate in all the grazing system, the slightly lower CH_4_ emission rate in OGR than in the pasture enclosures was attributed to the limited soil moisture content that inhibited the activity methanogens. The high CH_4_ emission during the wet season than during the dry season was also attributed to the differences in soil moisture content during the dry and wet seasons which affected the activity of soil methanogens. This is supported by the significant positive relationship between soil moisture and CH_4_ emission (*r*^*2*^ = 0.15, *P* < 0.001). The strong positive correlation between CH_4_ and CO_2_ fluxes (*r* = 0.54) imply that respiration was a confounding factor influencing methane production by creating anaerobic microsites for CH_4_ production. These observations reiterated studies which reported positive CH_4_ fluxes in tropical rangeland soils [77–79]. The positive relationship between CH_4_ flux and soil water content has been reported in previous studies in grassland soils [84, 85].

The average N_2_O flux rates in this study (18.6 μg N m^−2 ^h^−1^) were lower than those reported by Assouma et al. [26] in a semi-arid rangeland in Senegal (104.2 μg N m^−2 ^h^−1^), and comparable to fluxes recorded in smallholder farms in Kisumu County in Kenya (< 20 μg N m^−2 ^h^−1^) [16]. The observation that the N_2_O flux rate was similar in all the grazing systems suggests that the establishment of pasture enclosures have no influence on N_2_O emission, consistent with a study conducted in differently grazed semi-arid grasslands [72]. This could be the result of the higher soil bulk density in OGR and the high concentration of particulate organic matter in the enclosures [13]. The high bulk density created anaerobic microsites physically hence increasing the denitrification processes. On the other hand, the high concentration of particulate organic carbon promoted the consumption of O_2_ in the soil hence creating anoxic microsites with low redox potential. According to Christensen et al. [80] and Kuzyakov and Blagodatskaya [81], the denitrification processes in soil is associated with the amount and location of active organic carbon which promotes the consumption of O_2_. Therefore, the presence of anaerobic hotspots in both the OGR and in the enclosures could have contributed to the production of N_2_O in equal proportions. The soil N_2_O emissions exhibited a weak positive relationship with soil moisture (*r*^*2*^ = 0.10, *P* < 0.001), other studies reported that N_2_O emissions were insensitive to soil moisture [82]. This implies that soil moisture was the critical factor controlling N_2_O flux in semi-arid rangeland soils, likely because of the influence on mineral nitrogen and labile C [83, 84]. According to Bateman and Baggs [85], nitrification process dominates at WFPS between 35–60% and above 60% WFPS denitrification processes predominate in semiarid conditions. The WFPS in this study was generally below 60% suggesting that N_2_O was predominantly produced through the denitrification processes in the anaerobic microsites.

## Conclusions

This study demonstrates that the establishment of pasture enclosures in previously degraded grassland created a conducive environment which allowed the recovery of vegetation cover, aboveground biomass and surface soil properties like bulk density, organic carbon, and soil moisture retention. Consequently, the improved soil and vegetation conditions in the enclosures favored respiration processes in the soil that ultimately contributed to the enhanced emission of CO_2_ into the atmosphere, but did change emission patterns of CH_4_ and N_2_O. Soil moisture content played the key role in influencing the emission rates. However, the observed results in this study, together with reports indicating that enclosures can decrease ecosystem respiration and increase CH_4_ uptake in the soil, necessitate a long-term study to evaluate the patterns in annual CO_2_, N_2_O, CH_4_ fluxes from soils and determine the ecosystem carbon balance across the pastoral landscape in tropical rangelands.
